# Social Anxiety and Peer Relationships Predict Weight Recovery in Adolescent Onset Anorexia Nervosa

**DOI:** 10.1002/erv.70056

**Published:** 2025-12-06

**Authors:** Victoria Burmester, Erica Cini, Tahmida Baksh, Nikita Catalina Julius, Keziah Chung, Dasha Nicholls

**Affiliations:** ^1^ Division of Psychiatry Faculty of Medicine Imperial College London London UK; ^2^ Department of C&A Psychiatry IoPPN King's College London London UK; ^3^ Division of Medicine University College London London UK; ^4^ East London NHS Foundation Trust London UK

**Keywords:** adolescent, anorexia nervosa, interpersonal, peers, social anxiety, weight recovery

## Abstract

**Background:**

Adolescence is period of social change during which peer relationships are prioritised. The desire for social approval, which heavily influences behaviour, is often linked to an increased risk of developing an eating disorder (ED). Social cognition is impaired in ED and both autism and social anxiety frequently co‐occur. It is difficult to disentangle the contributing factors of cognitive and interpersonal difficulties in adolescents who present at children's ED services, and whether these relate to treatment response. We investigated whether baseline social measures predicted early treatment response after the initial 6‐month period.

**Methods:**

Adolescents aged 12–16 years (*N* = 983) treated by children's community ED services in England, UK, between 2017 and 2023, completed the Revised Child Anxiety and Depression Scales (RCADS) and/or the Strengths and Difficulties Questionnaire (SDQ) at assessment. Early weight restoration, defined as reaching at least 85% median‐BMI adjusted for age and sex, at 6 months, was the primary outcome. A multiple linear regression was conducted using three measures: RCADS' Social Phobia, SDQ's Peer Problems and SDQ's Prosocial Behaviour to predict the degree of early weight restoration (85%medBMI), which was the primary outcome.

**Results:**

The combined baseline social predictors: social phobia (standardised *β* = −0.382), peer problems (standardised *β* = −0.118) and prosocial behaviour (standardised *β* = −0.105) explained 20% of variance in weight restoration at 6‐months post assessment (*p* < 0.001). Individual predictors regressed to %mBMI, were not moderated by age, with peer problems negatively predicting weight at 6 months and prosociality scores demonstrating significant skew because self‐rated scores were high. Exploratory analyses showed that social anxiety was greater in those who did not reach early weight recovery at 6‐months post assessment.

**Conclusions:**

Assessing and addressing difficulties in social/peer group functioning might be a central consideration in treatment for AN in adolescents. Further research is needed to better understand the role of social functioning in recovery from AN.

## Highlights

- Baseline social functioning significantly predicts early weight recovery in adolescents with anorexia nervosa (AN): A combined model incorporating social anxiety, peer problems, and prosocial behaviour explained 20% of the variance in weight restoration at 6 months, with social anxiety and peer difficulties emerging as significant negative predictors.
- Peer problems independently and consistently predicted poorer weight outcomes: Across both combined and individual regression analyses, higher peer difficulties were associated with reduced likelihood of achieving early weight recovery, underscoring the importance of peer dynamics in treatment response.
- Prosocial behaviour showed a complex relationship with recovery: While higher prosociality was associated with better outcomes in the combined model, it paradoxically predicted lower weight gain when analysed independently, likely due to ceiling effects in self‐report measures, suggesting limitations in current assessment tools.

## 1 Introduction

Anorexia Nervosa (AN) has a high risk of mortality (Krug et al. 2025). Incidence increases steadily through adolescence, peaking at age 17 years, with an estimated incidence of 58 and 5 per 100,000 for young females and males, respectively (Petkova et al. 2019). Eating disorder (ED) prevalence has increased 7% year‐on‐year since 2009 (Beat 2015) and hospital admissions rose by 37% from 2017–2019 (NHS Digital 2019). These increases have been observed at a population level as well as in clinical services. In 2023, probable ED were identified in one in eight (12.5%) of 17‐ to 19‐year‐olds (Newlove‐Delgado et al. 2022). The UK's National Institute Health and Care Excellence recommends AN focussed family therapy (FT‐AN) as the first‐line treatment in young people, which is estimated as effective at achieving remission in around 40% of patients (Galmiche et al. 2019). However, FT‐AN does not directly address social functioning, unless impairment is severe or comorbid autism is diagnosed (Loomes and Bryant‐Waugh 2021). In AN, early weight restoration is the best predictor of successful remission (Van Huysse et al. 2020).

Adolescence is a critical period for the development of social competencies, as it coincides with the maturation of brain networks associated with social function (Bingham et al. 2011). During this stage, heightened self‐consciousness, complex thinking styles, and evolving interpersonal dynamics make adolescents particularly susceptible to social challenges (Blakemore 2008; Orben et al. 2020). These challenges are of particular interest in understanding the vulnerabilities that predispose adolescents to ED, such as AN, since social bonds protect against ED development (Keel et al. 2001; Watson et al. 2016). Peer interactions play a vital role during adolescence and strong friendships protect against ED development. Altered brain responses in social circuits can amplify reward‐seeking behaviours related to social desirability, particularly with regard to appearance ideals, contributing to the vulnerabilities associated with AN (Prince et al. 2022). Interpersonal difficulties mediate between childhood emotion regulation and adolescent disordered eating, emphasising the importance of peer dynamics (Krug et al. 2013; Smink et al. 2018). The ability to navigate social interactions and need for peer approval may influence ED onset and recovery (Warne et al. 2023).

Social difficulties in AN manifest in various ways, ranging from treatable conditions, such as reduced empathy in starvation, to communication challenges due to neurodiversity as seen in autistic individuals, who are especially vulnerable to AN (Schaumberg et al. 2021). Social anxiety is highly prevalent among adolescents with ED, often predating their onset, and associated with the worsening of symptoms (Kerr‐Gaffney et al. 2018). Longitudinal studies identify social anxiety as a predictor of illness development (McClelland et al. 2020; Schaumberg et al. 2019). However, the relationship between social anxiety and peer problems with recovery trajectories in adolescents with AN remains underexplored.

In contrast to the challenges posed by social anxiety and peer difficulties, prosocial behaviours that benefit others offer a potential area of resilience. Prosociality is closely linked to the development of understanding others' emotions, intentions, and social norms, which are supported by regions such as the medial prefrontal cortex and temporoparietal junction that mature rapidly during adolescence. Experimentally, adolescents with an ED showed reduced prosocial behaviour in a computerised task than age‐matched controls without an ED (Rowlands et al. 2023). These skills may mitigate the psychosocial impairments associated with ED.

There is compelling evidence that social function and competence factors, such as social anxiety, peer problems, and prosocial behaviour, interact in complex ways (Chiu et al. 2021), suggesting they should not be examined in isolation. Despite mounting evidence of reciprocal associations between social functioning and ED symptoms (Smink et al. 2018; Stern et al. 2023), and evidence that psychosocial factors predict onset (Stice et al. 2017; Stice and Van Ryzin 2019), the impact of social function on recovery adolescents with AN has not been investigated in clinical populations. This study addressed this gap by investigating whether a model with three different areas of social functioning could predict weight recovery at 6 months in adolescents undergoing community treatment for AN. Understanding their combined predictive value could provide novel insights into tailoring interventions to support adolescents with AN.

## 2 Method and Materials

### 2.1 Participants

The sample was 983 patients aged 12–16 years who completed an initial six months of weekly ED‐focussed family therapy as outpatients in a community specialist ED service between 2017–2023. Data were obtained from seven National Health Service (NHS) ED clinics in the northwest, southwest, and southeast of England, UK. For this analysis, AN patients, both binge and restrictive subtypes, with grade 1 thinness or below, determined using age‐ and sex‐specific cut‐offs for children corresponding to a BMI of 18.5 kg/m^2^ at age 18 years (Cole et al. 1995). Patients whose records lacked 6‐month data were excluded.

### 2.2 Measures

#### 2.2.1 Body Mass Index

Early weight recovery: Weight and height were measured by clinicians at ED clinics and adjusted for age and sex by using percentage median Body Mass Index (%mBMI) using the formula BMI/50^th^ percentile BMI for age and sex*100 (Cole et al. 1995). The %mBMI used for the outcome was that recorded at, or closest to, 6 months after assessment. Subscales that related to social functioning were selected from mental health questionnaires routinely completed at assessment in children's ED services.

The Revised Child Anxiety and Depression Scales (RCADS) is a 47‐item measure designed to assess symptoms corresponding to anxiety disorders and depression in 8–18‐year‐olds. The measure includes six subscales: Separation Anxiety Disorder, Social Phobia, Generalised Anxiety Disorder, and panic disorder and in this study, only the nine questions of the Social Phobia subscale (Chorpita et al. 2015) were used. Respondents rate the frequency of statements relating to social anxiety as occurring never, sometimes, often, and always. Scores range from 0–27, with higher scores indicating higher social anxiety.

The self‐reported 25‐item Strengths and Difficulties Questionnaire (SDQ) is a behavioural screening tool for 11–17‐year‐olds (Goodman 1997). It comprises five subscales: (1) Emotional Symptoms, (2) Conduct Problems (3) Hyperactivity/Inattention (4) Peer Problems and (5) Prosocial Behaviour. Higher scores indicate increasing problems, except for the prosocial scale, which is reversed. In this report we used the Peer Problems and Prosocial Behaviour subscales.

#### 2.2.2 Procedure

The study was approved by NHS Health Research Authority (Ref:308181) as secondary data analysis and sponsored by Imperial College London. Patient weight and height, routinely collected at assessment and 6 months, and psychological measures were anonymised by ED clinics before sharing and analysis.

#### 2.2.3 Data

Data were analysed using SPSS v29 and for the moderation analysis PROCESS v4.2 was also used (Hayes 2017). A power calculation using 0.95 power and a conservative medium effect *f* ^2^ = 0.15 reflecting social cognition studies in adolescent ED populations (McFillin et al. 2012; Tomova et al. 2020) determined a sample size of *n* = 107 was required. Although SDQ, and RCADS scores were requested from individual NHS Trusts, which oversee the UK's regional NHS healthcare provision, the available data varied considerably due to data loss, COVID‐related systems backlogs, access difficulties, or local variations in outcome collection practices. Additionally, Trusts do not receive payments to support participation in secondary analysis research, so data were obtained on a goodwill basis.

To test the hypothesis that social phobia, prosocial behaviour and peer problems, would together predict early weight recovery, data from a sample of patients achieving early weight recovery at 6 months who also had full baseline social data at assessment (*n* = 270) were analysed. The primary outcome was absolute %mBMI at 6 months. To assess whether baseline social scores would predict %mBMI in those who achieved weight recovery but had incomplete baseline social measures, social anxiety, peer problems and prosociality were assessed independently with three linear regressions. Age was included as a moderator in each model to explore whether it affected the predictive relationships between social functioning and weight recovery. Three independent *t* tests examining each predictor individually explored mean differences in social functioning between those who had and those who had not reached weight recovery at 6 months.

## 3 Results

The full sample of patients with ED below Grade 1 thinness (*N* = 983) was predominantly female (male, *n* = 18) and comprised those with both restrictive and binge‐purge AN subtypes. The majority were of white ethnicity, except in urban areas, which were ethnically heterogeneous. The clinical characteristics of the subsample (*n* = 270) used to address our hypothesis, who had reached early weight recovery at 6 months and completed both the SDQ and RDQ at baseline are reported together with the full sample in Table 1.

**TABLE 1:** Descriptive statistics and clinical measures at baseline and 6 months.

Baseline assessment	Full sample (*N* = 983)	EWR sample (*n* = 668)	EWR sample with RCADS & SDQ at baseline (*n* = 270)
Age (SD)	15.28 (0.861)	15.25 (0.901)	15.61 (0.646)
Ethnicity			
Asian	9%	8.9%	4.4%
Black	1%	1.2%	0.7%
Mixed	7.2%	4.8%	3.7%
White	69.9%	68.6%	90.4%
Other	4.3%	5.1%	0.7%
Missing	8%	10.9%	—
%mBMI age/sex adjusted (SD)	82.21 (7.09)	83.17 (6.25)	84.07 (4.76)
Missing data	*n* = 642	*n* = 376	*n* = 233
RCADS social phobia (SD)	20.61 (4.97)	19.57 (5.30)	18.97 (4.09)
Missing data	*n* = 174	*n* = 153	—
SDQ peer problems (SD)	3.11 (1.65)	3.15 (1.69)	3.30 (1.66)
Missing data	*n* = 450	*n* = 276	—
SDQ prosocial behaviour (SD)	(*n* = 539), 8.30 (1.82)	8.38 (1.79)	8.61 (1.59)
Missing data	*n* = 444	*n* = 152	—

**Six months**	**Mean (SD)**	**Mean (SD)**	**Mean (SD)**
%mBMI age/sex adjusted (SD)	92.37 (8.11)	92.37 (8.10)	90.04 (4.29)
Missing data	*n* = 327	None	None

Abbreviations: EWR, early weight recovery (> 85%mBMI at 6 months); RCADS, Revised Child Anxiety and Depression Scale; SDQ, Strengths and Difficulties Questionnaire.

Out of a sample of 983 children with AN, just over two‐thirds (*n* = 668) achieved early‐weight recovery at six months, of whom *n* = 515 completed baseline RCADS, *n* = 392 completed SDQ, and *n* = 270 completed both questionnaires.

### 3.1 Multiple Linear Regression Predicting Weight Recovery Using Combined Social Measures

Pearson coefficient correlations among the three predictor variables were low: RCADS social phobia and peer problems *r* = ‒ 0.14, RCADS social phobia and prosocial at *r* = 0.33, and prosocial versus peer problems *r* = 0.15. Among patients who reached early weight recovery and completed all three baseline measures, a multiple regression with RCADS social phobia, SDQ prosocial behaviour & SDQ peer problems significantly predicted early weight recovery at 6 months (*p* = 0.001) explaining 20% (adjusted *r* ^2^ = 0.20) of the variance in %mBMI gain, see Table 2 for individual predictors.

**TABLE 2:** Multiple linear regression predicting %mBMI at 6 months, *n* = 270.

Predictor	*β* (standardised)	*t*	*p*‐value
RCADS social phobia	−0.382	6.53	0.001
SDQ peer problems	−0.118	2.14	0.020
SDQ prosocial behaviour	−0.105	1.82	0.070

Abbreviations: RCADS, Revised Child Anxiety and Depression Scale; SDQ, Strengths and Difficulties Questionnaire.

### 3.2 Simple Linear Regressions Predicting Weight Gain Using Separate Social Measures

Using only data from patients who achieved early‐weight recovery, each social predictor was regressed individually to assess their independent contributions to %mBMI at 6 months, see Table 3. Over half the sample (53%) completed RCADS social phobia, which remained a negative predictor of weight recovery but was not significant by itself in predicting weight gain. SDQ data were available from 40% of the full sample and peer problems remained a significant negative predictor of weight recovery. However, as a single predictor of weight outcome, prosocial behaviour reversed direction, and lower scores predicted increasing weight. However, when regressed individually the variance each predictor explained was low, in contrast to the large amount of variance the three predictors explained when combined.

**TABLE 3:** Single linear regression analyses predicting %mBMI at 6 months.

Predictor	*n*	*β* (standardised)	*t*	*p*‐value	*R* ^2^
RCADS social phobia	515	−0.056	1.26	0.207	0.003
SDQ peer problems	392	−0.152	3.31	0.001	0.023
SDQ prosocial behaviour	393	−0.220	3.69	0.003	0.048

Abbreviations: RCADS, Revised Child Anxiety and Depression Scale; SDQ, Strengths and Difficulties Questionnaire.

### 3.3 Exploratory Analyses

#### 3.3.1 Moderation by Age

Using the subsample who achieved early weight recovery, age was incorporated as a moderator into the separate regressions but did not moderate the relationships between early‐weight recovery at 6 months and RCADS social phobia (*p* = 0.247) or SDQ peer problems (*p* = 0.764) or prosocial behaviour (*p* = 0.340).

#### 3.3.2 Comparison of Baseline Social Measures by Early Weight Recovery Status

An independent *t* test explored the differences between SDQ peer problems in those who did and did not achieve early weight recovery, see Table 4. A Mann Whitney‐U analysis was conducted for RCADS social phobia scores (*n* = 803), which were negatively skewed with positive kurtosis, and for SDQ prosocial behaviour scores (*n* = 539) that were negatively skewed. Social anxiety was higher in those who did not reach 85%mBMI at 6 months, and prosociality was higher in the recovery group. There were no significant differences in peer problems between weight‐recovered patients and those who were still underweight at 6 months.

**TABLE 4:** Comparison of baseline scores by early weight recovery status.

Measure	*n* (total)	Mean (SD)		Statistic (*Z*/*t*)	*p*‐value	Effect (*r*/*d*)
RCADS Social phobia	803	339 (mean rank)		*Z* = −10.32	< 0.001	*r* = 0.36
EWR	515	19.57 (5.30)				
No EWR	288	22.63 (3.34)				
SDQ peer problems	532			*t* (530) = 1.06	0.290	*d* = 0.10
EWR	392	3.15 (1.70)				
No EWR	140	2.98 (1.52)				
SDQ prosocial behaviour	538	276 (mean rank)	251	*Z* = −1.93	0.053	*r* = 0.08
EWR	393	8.38 (1.79)				
No EWR	145	8.08 (1.90)				

Abbreviations: EWR, early weight recovery (> 85%mBMI at 6 months); RCADS, Revised Child Anxiety and Depression Scale; SDQ, Strengths and Difficulties Questionnaire.

## 4 Discussion

To our knowledge, this is the first longitudinal evidence using clinical data from a large sample of adolescents being treated for AN, demonstrating that social functioning at assessment predicts early‐weight restoration. In patients who achieved early‐weight recovery. A model using peer problems, social anxiety, and prosocial behaviour as predictors, explained 20% of the variance in %mBMI at 6 months post assessment. Peer problems and social anxiety were significant negative predictors, whereas prosocial behaviour positively predicted weight recovery, but did not reach significance. When explored separately as predictors of %mBMI in early weight recovered patients, social anxiety did not significantly predict weight gain but peer problems and, unexpectedly, prosociality were significant independent negative predictors of weight gain. These findings are in line with cross‐sectional and nonclinical studies reporting associations between social function and EDs (Ganson et al. 2025; Lotery et al. 2024; Marffy et al. 2024; McFillin et al. 2012; Mikami and Hinshaw 2006; Scully et al. 2023; Stern et al. 2025; Uzunian and Vitalle 2015).

Whilst weight recovery does not constitute recovery from AN, re‐feeding rapidly reverses cognitive inefficiencies resulting from malnutrition and consistently predicts full remission (Doyle et al. 2010; Hartmann et al. 2007; Lock et al. 2006, 2010; Madden et al. 2015; Van Huysse et al. 2020). Since prolonged food deprivation results in heightened anxiety, low mood, and social withdrawal (Spettigue et al. 2025), uniformly high levels of social anxiety across the entire sample might be expected. In this sample of adolescent patients with AN, social anxiety levels were high but nonetheless, for those who achieved weight recovery after 6 months of treatment, social anxiety was significantly lower, which suggests a role for individual differences in social anxiety in determining treatment progress. Despite baseline peer problems being low and not differing between individuals who did and did not achieve early‐weight restoration 6 months later, increased difficulties with peers significantly predicted weight gain in combined and single regression models. As expected, prosocial behaviour positively predicted weight gain, albeit nonsignificantly, with social anxiety and peer problems included in the regression model. However, as a single predictor prosociality reversed and negatively predicted %mBMI. This was likely an artifact of the large ceiling effect whereby most participants rated themselves as highly prosocial limiting the model's ability to distinguish among high scorers.

Age did not moderate the relationship between any social measures and %mBMI in those reaching early weight recovery. However, in adolescents, pubertal status may be a more reliable and appropriate measure of development than chronological age, since brain maturation is highly dependent on sex hormones. The well‐supported hypothesis of dual systems development during adolescence (Steinberg 2010), highlights how frontal networks concerned with human cognition, including decision‐making, inhibition, and abstract reasoning, develop independently of subcortical regions associated with reward‐seeking and affect‐processing (Casey et al. 2008; Somerville et al. 2010; Steinberg 2017). This results in a developmental mismatch where one area develops before the other; typically, limbic areas associated with reward develop ahead of frontal networks associated with inhibition.

Adolescence is characterised by fluctuations and changes in multiple and overlapping domains such as, impulse control, risk taking, emotional regulation, peer reward, social awareness, inhibition, abstract reasoning, and social emotions (Reynolds et al. 2013). During adolescence, top‐down control of negative affective states, such as fear, anxiety, or low mood, may deviate from the executive functioning of a mature brain (Graber and Petersen 1991). Inappropriate or unhelpful coping strategies often emerge during adolescence, particularly when linked to peer approval, and transfer of attachment to peers may not be successful without secure primary bonds from childhood (Masten et al. 2009).

The sample was predominantly white, with increased ethnic heterogeneity in urban areas. Aside from mounting evidence that culture and ethnicity shape ED phenotypes (Cheng et al. 2019; Misra et al. 2009), and clinical reports of amenorrhoea onset varying by ethnicity, individualising a healthy weight using biological factors such as the resumption of menses in post‐menarcheal females is important (Aruna et al. 2024). Further cross‐cultural research would not only increase diagnostic accuracy, but potentially influence recovery, as weight recovery thresholds are important motivators that can provide valuable direction, especially during the flux of adolescence.

Although the present study found strong evidence that baseline social functioning was predictive of weight recovery and aligns with adult clinical data (Stern et al. 2025), one‐off self‐report scores may not be reliable, particularly in a population with AN. Clinically, self‐report is typically coupled with parent‐report to obtain a comprehensive view on functioning. However, parents may not always be well placed to assess social anxiety and problems with peer relationships, as adolescents may not share information about friendships with parents, and task‐based or objective measurement of social function should be considered.

ED prevalence in males is rising, and further research could profitably examine differences in social functioning by sex and/or gender, which become pronounced during puberty (Gorrell and Murray 2019). Research is needed to develop measures sensitive enough to identify nuanced psychosocial difficulties, such as cognitive biases, which could amplify, consolidate, or impede recovery. Developing age‐appropriate and objective instruments to measure adolescent social function would also facilitate stratification of the overlapping subpopulations of young people with AN, for example those with comorbid autism, to identify their unique contributions to ED development and outcomes (Westwood and Tchanturia 2017).

A strength and a limitation of this study is that it combines outcomes from geographically diverse regions. Trusts from ethnically diverse, high density urban areas where demand for clinical care outweighs supply, and from rural areas with lower population densities and less social diversity, were considered. There is evidence that the quality of care received from services situated in rural areas differs considerably from those in metropolitan cities with high demand and long waiting lists (Brown et al. 2025). However, the study does not account for other explanatory factors that may impact patient outcomes, such as staff training and experience. Adherence to best practice guidelines varies across Trusts, and so too the systems of recording patient data. Some Trusts do not administer or record baseline social function and, in addition, the data were requested from Trusts during the transition back to in‐person treatment at the end of the COVID‐19 pandemic, a period that resulted in treatment disruption or adaptation and escalating demand. For many Trusts, the pandemic provided an opportunity to focus on, evaluate and re‐structure existing systems. The additional burden on returning staff to familiarise themselves with changes inevitably reduced their capacity for research activities. This resulted in a smaller sample than anticipated that provided self‐report social measures and a self‐report ED metric at 6 months. Our outcome was, therefore, limited to weight recovery rather than improvement in psychological function as the primary outcome as only a small number of services were able to provide both social measures at baseline.

## 5 Conclusion

In this large sample of young people presenting with low weight AN for clinical care, a clear pattern was seen whereby better baseline social function predicted weight recovery, independent of age. Assessing and addressing difficulties in social/peer group functioning might be a central consideration in treatment for AN in adolescents and adolescents with AN might benefit from interventions to improve social functioning both to promote weight recovery and help social re‐integration at discharge. Further research is needed to better understand the role of social functioning in recovery from AN.

## Author Contributions

V.B. and D.N. designed the study. V.B., N.C.J., E.C., T.B., K.C. collected data. V.B., K.C. and N.C.J. analysed data, V.B. wrote the first draft, all authors contributed to the final draft.

## Funding

This report is independent research funded by the Rosetrees Trust.

## Ethics Statement

This study was approved by the NHS Health Research Authority (Ref: 308181) as a secondary data analysis of anonymised clinical records. All data were collected as part of routine clinical care and shared in accordance with NHS data governance protocols. No identifiable patient information was used, and consent for secondary use was obtained via service‐level agreements with participating NHS Trusts.

## Conflicts of Interest

The authors declare no conflicts of interest.

## Data Availability

All data are available from the authors upon reasonable request.

## References

[erv70056-bib-0001] Aruna, R. , P. Muruganandam , and S. Niveatha . 2024. “Eating Habit, Body Image, and Gender–Is There Any association?–A Comparative Study Among Medical Students From Southern India.” Journal of Education and Health Promotion 13, no. 1: 326. 10.4103/jehp.jehp_72_24. 39429832 PMC11488783

[erv70056-bib-0002] Beat, U. K. 2015. The Costs of Eating Disorders: Social, Health and Economic Impacts: Norwich: Author, 237.

[erv70056-bib-0003] Bingham, B. , K. McFadden , X. Zhang , S. Bhatnagar , S. Beck , and R. Valentino . 2011. “Early Adolescence as a Critical Window During Which Social Stress Distinctly Alters Behavior and Brain Norepinephrine Activity.” Neuropsychopharmacology 36, no. 4: 896–909. 10.1038/npp.2010.229. 21178981 PMC3055730

[erv70056-bib-0004] Blakemore, S. J. 2008. “The Social Brain in Adolescence.” Nature Reviews Neuroscience 9, no. 4: 267–277. 10.1038/nrn2353. 18354399

[erv70056-bib-0005] Brown, R. , C. Murphy‐Morgan , J. Downs , and D. Branley‐Bell . 2025. “A Call for Strategy on Eating Disorders: The Need for a Comprehensive Eating Disorder Strategy in England and Specific Guidance for the Remote Delivery of Eating Disorder Services.” Journal of Eating Disorders 13, no. 1: 54. 10.1186/s40337-025-01224-y. 40140927 PMC11948745

[erv70056-bib-0006] Casey, B. J. , S. Getz , and A. Galvan . 2008. “The Adolescent Brain.” Developmental review 28, no. 1: 62–77. 10.1016/j.dr.2007.08.003. 18688292 PMC2500212

[erv70056-bib-0007] Cheng, Z. H. , V. L. Perko , L. Fuller‐Marashi , J. M. Gau , and E. Stice . 2019. “Ethnic Differences in Eating Disorder Prevalence, Risk Factors, and Predictive Effects of Risk Factors Among Young Women.” Eating Behaviors 32: 23–30. 10.1016/j.eatbeh.2018.11.004. 30529736 PMC6382562

[erv70056-bib-0008] Chiu, K. , D. M. Clark , and E. Leigh . 2021. “Cognitive Predictors of Adolescent Social Anxiety.” Behaviour Research and Therapy 137: 103801. 10.1016/j.brat.2020.103801. 33421893 PMC7846721

[erv70056-bib-0009] Chorpita, B. F. , C. Ebesutani , and S. H. Spence . 2015. “Revised Children’s Anxiety and Depression Scale.” Hämtad från. https://www.childfirst.ucla.edu/wpcontent/uploads/sites/163/2018/03/RCADSUsersGuide20150701.pdf.

[erv70056-bib-0010] Cole, T. J. , J. V. Freeman , and M. A. Preece . 1995. “Body Mass Index Reference Curves for the UK, 1990.” Archives of Disease in Childhood 73, no. 1: 25–29. 10.1136/adc.73.1.25. 7639544 PMC1511150

[erv70056-bib-0011] Doyle, P. M. , D. Le Grange , K. Loeb , A. C. Doyle , and R. D. Crosby . 2010. “Early Response to Family‐Based Treatment for Adolescent Anorexia Nervosa.” International Journal of Eating Disorders 43, no. 7: 659–662. 10.1002/eat.20764. 19816862 PMC8693442

[erv70056-bib-0012] Galmiche, M. , P. Déchelotte , G. Lambert , and M. P. Tavolacci . 2019. “Prevalence of Eating Disorders over the 2000‐2018 Period: A Systematic Literature Review.” American Journal of Clinical Nutrition 109, no. 5: 1402–1413. 10.1093/ajcn/nqy342. 31051507

[erv70056-bib-0013] Ganson, K. T. , K. Cuccolo , and J. M. Nagata . 2025. “Loneliness Is Associated With Eating Disorders Among a National Sample of US College Students During the COVID‐19 Pandemic.” Journal of American College Health 73, no. 2: 462–466. 10.1080/07448481.2023.2232872. 37486743 PMC11384234

[erv70056-bib-0014] Goodman, R. 1997. “The Strengths and Difficulties Questionnaire: A Research Note.” Journal of Child Psychology and Psychiatry 38, no. 5: 581–586. 10.1111/j.1469-7610.1997.tb01545.x. 9255702

[erv70056-bib-0015] Gorrell, S. , and S. B. Murray . 2019. “Eating Disorders in Males.” Child and Adolescent Psychiatric Clinics of North America 28, no. 4: 641–651. 10.1016/j.chc.2019.05.012. 31443881 PMC6785984

[erv70056-bib-0016] Graber, J. A. , and A. C. Petersen . 1991. “Cognitive Changes at Adolescence: Biological Perspectives.” Brain Maturation and Cognitive Development: Comparative and Cross‐Cultural Perspectives: 253–279.

[erv70056-bib-0017] Hartmann, A. , C. Wirth , and A. Zeeck . 2007. “Prediction of Failure of Inpatient Treatment of Anorexia Nervosa From Early Weight Gain.” Psychotherapy Research 17, no. 2: 218–229. 10.1080/10503300600702315.

[erv70056-bib-0018] Hayes, A. F. 2017. Introduction to Mediation, Moderation, and Conditional Process Analysis: A Regression‐based Approach. Guilford publications.

[erv70056-bib-0019] Keel, P. K. , G. R. Leon , and J. A. Fulkerson . 2001. “Vulnerability to Eating Disorders in Childhood and Adolescence.” In Vulnerability to Psychopathology: Risk Across the Lifespan, edited by R. E. Ingram and J. M. Price , 389–411. Guilford Press.

[erv70056-bib-0020] Kerr‐Gaffney, J. , A. Harrison , and K. Tchanturia . 2018. “Social Anxiety in the Eating Disorders: A Systematic Review and Meta‐Analysis.” Psychological Medicine 48, no. 15: 2477–2491. 10.1017/s0033291718000752. 29631640

[erv70056-bib-0021] Krug, I. , S. Liu , J. Portingale , et al. 2025. “A Meta‐Analysis of Mortality Rates in Eating Disorders: An Update of the Literature From 2010 to 2024.” Clinical Psychology Review 116: 102547. 10.1016/j.cpr.2025.102547. 39889307

[erv70056-bib-0022] Krug, I. , E. Penelo , F. Fernandez‐Aranda , et al. 2013. “Low Social Interactions in Eating Disorder Patients in Childhood and Adulthood: A Multi‐Centre European Case Control Study.” Journal of Health Psychology 18, no. 1: 26–37. 10.1177/1359105311435946. 22491496

[erv70056-bib-0023] Lock, J. , J. Couturier , S. Bryson , and S. Agras . 2006. “Predictors of Dropout and Remission in Family Therapy for Adolescent Anorexia Nervosa in a Randomized Clinical Trial.” International Journal of Eating Disorders 39, no. 8: 639–647. 10.1002/eat.20328. 16927385

[erv70056-bib-0024] Lock, J. , D. Le Grange , W. S. Agras , A. Moye , S. W. Bryson , and B. Jo . 2010. “Randomized Clinical Trial Comparing Family‐Based Treatment With Adolescent‐Focused Individual Therapy for Adolescents With Anorexia Nervosa.” Archives of General Psychiatry 67, no. 10: 1025–1032. 10.1001/archgenpsychiatry.2010.128. 20921118 PMC3038846

[erv70056-bib-0025] Loomes, R. , and R. Bryant‐Waugh . 2021. “Widening the Reach of Family‐Based Interventions for Anorexia Nervosa: Autism‐adaptations for Children and Adolescents.” Journal of eating disorders 9: 1–11. 10.1186/s40337-021-00511-8. 34863292 PMC8645124

[erv70056-bib-0026] Lotery, E. , R. Bell , G. Combe , L. Biddle , and H. Bould . 2024. “Qualitative Study of the Impact on Recovery of Peer Relationships Between Female Inpatients During Treatment for Anorexia Nervosa in the United Kingdom.” International Journal of Eating Disorders 57, no. 2: 353–362. 10.1002/eat.24102. 38062886

[erv70056-bib-0027] Madden, S. , J. Miskovic‐Wheatley , A. Wallis , M. Kohn , P. Hay , and S. Touyz . 2015. “Early Weight Gain in Family‐Based Treatment Predicts Greater Weight Gain and Remission at the End of Treatment and Remission at 12‐month Follow‐Up in Adolescent Anorexia Nervosa.” International Journal of Eating Disorders 48, no. 7: 919–922. 10.1002/eat.22414. 26488111

[erv70056-bib-0028] Marffy, M. J. , J. Fox , and M. Williams . 2024. “An Exploration of the Relationship Between Loneliness, the Severity of Eating Disorder‐Related Symptoms and the Experience of the ‘Anorexic Voice’.” Psychology and Psychotherapy: Theory, Research and Practice 97, no. 1: 122–137. 10.1111/papt.12502. 37792343

[erv70056-bib-0029] Masten, C. L. , J. Juvonen , and A. Spatzier . 2009. “Relative Importance of Parents and Peers: Differences in Academic and Social Behaviors at Three Grade Levels Spanning Late Childhood and Early Adolescence.” Journal of Early Adolescence 29, no. 6: 773–799. 10.1177/0272431608325504.

[erv70056-bib-0030] McClelland, J. , L. Robinson , R. Potterton , V. Mountford , and U. Schmidt . 2020. “Symptom Trajectories into Eating Disorders: A Systematic Review of Longitudinal, Nonclinical Studies in Children/Adolescents.” European Psychiatry 63, no. 1: e60. 10.1192/j.eurpsy.2020.55. 32450945 PMC7355161

[erv70056-bib-0031] McFillin, R. K. , S. C. Cahn , V. S. Burks , M. P. Levine , S. L. Loney , and R. L. Levine . 2012. “Social Information‐Processing and Coping in Adolescent Females Diagnosed With an Eating Disorder: Toward a Greater Understanding of Control.” Eating Disorders 20, no. 1: 42–59. 10.1080/10640266.2012.635565. 22188059

[erv70056-bib-0032] Mikami, A. Y. , and S. P. Hinshaw . 2006. “Resilient Adolescent Adjustment Among Girls: Buffers of Childhood Peer Rejection and Attention‐Deficit/Hyperactivity Disorder.” Journal of Abnormal Child Psychology 34, no. 6: 823–837. 10.1007/s10802-006-9062-7. PMC2935279 17051436

[erv70056-bib-0033] Misra, A. , P. Chowbey , B. M. Makkar , et al. and Consensus Group . 2009. “Consensus Statement for Diagnosis of Obesity, Abdominal Obesity and the Metabolic Syndrome for Asian Indians and Recommendations for Physical Activity, Medical and Surgical Management.” Journal of the Association of Physicians of India 57, no. 2: 163–170. PMID: 19582986. 19582986

[erv70056-bib-0034] Newlove‐Delgado, T. , Marcheselli, F. , Williams, T. , et al. 2022. Mental Health of Children and Young People in England, 2022‐Wave 3 Follow Up to the 2017 Survey.

[erv70056-bib-0035] NHS Digital . 2019. “Hospital Admissions for Eating Disorders.” Accessed 08 15, 20. https://digital.nhs.uk/data‐and‐information/find‐data‐and‐publications/supplementary‐information/2019‐supplementary‐information‐files/hospital‐admissions‐for‐eating‐disorders.

[erv70056-bib-0036] Orben, A. , L. Tomova , and S. J. Blakemore . 2020. “The Effects of Social Deprivation on Adolescent Development and Mental Health.” Lancet Child & Adolescent Health 4, no. 8: 634–640. 10.1016/s2352-4642(20)30186-3. 32540024 PMC7292584

[erv70056-bib-0037] Petkova, M. S. , D. Nicholls , T. Ford , et al. 2019. “Incidence of Anorexia Nervosa in Young People in the UK and Ireland: A National Surveillance Study.” BMJ Open 9, no. 10: e027339. 10.1136/bmjopen-2018-027339. PMC6954494 31640991

[erv70056-bib-0038] Prince, T. , L. McLoughlin , J. Lagopoulos , R. Elwyn , and D. F. Hermens . 2022. “The Neural Correlates of Socio‐Cognitive Factors and Eating Disorders in Young People: A Systematic Review.” Journal of Psychiatric Research 156: 647–659. 10.1016/j.jpsychires.2022.10.058. 36375232

[erv70056-bib-0039] Reynolds, E. K. , W. M. Schreiber , K. Geisel , L. MacPherson , M. Ernst , and C. W. Lejuez . 2013. “Influence of Social Stress on Risk‐Taking Behavior in Adolescents.” Journal of Anxiety Disorders 27, no. 3: 272–277. 10.1016/j.janxdis.2013.02.010. 23602940 PMC3693744

[erv70056-bib-0040] Rowlands, K. , M. Simic , J. Treasure , and V. Cardi . 2023. “Emotional Reactivity and Prosocial Behaviour in Response to Witnessing Social Exclusion in Adolescents With Eating Disorders and Healthy Controls.” Journal of Eating Disorders 11, no. 1: 224. 10.1186/s40337-023-00927-4. 38098100 PMC10722719

[erv70056-bib-0041] Schaumberg, K. , S. Zerwas , E. Goodman , Z. Yilmaz , C. M. Bulik , and N. Micali . 2019. “Anxiety Disorder Symptoms at Age 10 Predict Eating Disorder Symptoms and Diagnoses in Adolescence.” Journal of Child Psychology and Psychiatry 60, no. 6: 686–696. 10.1111/jcpp.12984. 30353925 PMC6482103

[erv70056-bib-0042] Schaumberg, K. , S. C. Zerwas , C. M. Bulik , C. Fiorentini , and N. Micali . 2021. “Prospective Associations Between Childhood Social Communication Processes and Adolescent Eating Disorder Symptoms in an Epidemiological Sample.” European Child & Adolescent Psychiatry 30, no. 12: 1929–1938. 10.1007/s00787-020-01655-9. 33064208 PMC8050127

[erv70056-bib-0043] Scully, M. , L. Swords , and E. Nixon . 2023. “Social Comparisons on Social Media: Online Appearance‐Related Activity and Body Dissatisfaction in Adolescent Girls.” Irish Journal of Psychological Medicine 40, no. 1: 31–42. 10.1017/ipm.2020.93. 32912367

[erv70056-bib-0044] Smink, F. R. , D. van Hoeken , J. K. Dijkstra , M. Deen , A. J. Oldehinkel , and H. W. Hoek . 2018. “Self‐Esteem and Peer‐Perceived Social Status in Early Adolescence and Prediction of Eating Pathology in Young Adulthood.” International Journal of Eating Disorders 51, no. 8: 852–862. 10.1002/eat.22875. 29704262 PMC6282973

[erv70056-bib-0045] Somerville, L. H. , R. M. Jones , and B. J. Casey . 2010. “A Time of Change: Behavioral and Neural Correlates of Adolescent Sensitivity to Appetitive and Aversive Environmental Cues.” Brain and Cognition 72, no. 1: 124–133. 10.1016/j.bandc.2009.07.003. 19695759 PMC2814936

[erv70056-bib-0046] Spettigue, W. , S. Drouin , L. Isserlin , et al. 2025. “The Psychological, Cognitive, and Behavioural Effects of Starvation in Humans: A Scoping Review.” European Eating Disorders Review 33, no. 4: 666–690. 10.1002/erv.3174. 39887591

[erv70056-bib-0047] Steinberg, L. 2010. “A Dual Systems Model of Adolescent Risk‐Taking.” Developmental Psychobiology: The Journal of the International Society for Developmental Psychobiology 52, no. 3: 216–224. 10.1002/dev.20445. 20213754

[erv70056-bib-0048] Steinberg, L. 2017. “A Social Neuroscience Perspective on Adolescent Risk‐Taking.” In Biosocial Theories of Crime, 435–463. Routledge.

[erv70056-bib-0049] Stern, M. , P. Rohde , C. D. Desjardins , J. S. Perry , and E. Stice . 2025. “Prospective Reciprocal Relations Between Psychosocial Impairment and Eating Disorder Symptoms in a High‐Risk Sample.” Eating Behaviors 57: 101958. 10.1016/j.eatbeh.2025.101958. 39955955

[erv70056-bib-0050] Stern, M. , L. Rubino , C. Desjardins , and E. Stice . 2023. “Prospective Reciprocal Relations Between Social Support and Eating Disorder Symptoms.” Journal of Psychopathology and Clinical Science 132, no. 8: 1043–1050. 10.1037/abn0000861. 38010772 PMC10683857

[erv70056-bib-0051] Stice, E. , J. M. Gau , P. Rohde , and H. Shaw . 2017. “Risk Factors That Predict Future Onset of Each DSM–5 Eating Disorder: Predictive Specificity in High‐Risk Adolescent Females.” Journal of Abnormal Psychology 126, no. 1: 38–51. 10.1037/abn0000219. 27709979 PMC5215960

[erv70056-bib-0052] Stice, E. , and M. J. Van Ryzin . 2019. “A Prospective Test of the Temporal Sequencing of Risk Factor Emergence in the Dual Pathway Model of Eating Disorders.” Journal of Abnormal Psychology 128, no. 2: 119–128. 10.1037/abn0000400. 30570269 PMC6361717

[erv70056-bib-0053] Tomova, L. , K. L. Wang , T. Thompson , et al. 2020. “Acute Social Isolation Evokes Midbrain Craving Responses Similar to Hunger.” Nature Neuroscience 23, no. 12: 1597–1605. 10.1038/s41593-020-00742-z. 33230328 PMC8580014

[erv70056-bib-0054] Uzunian, L. G. , and M. S. D. S. Vitalle . 2015. “Social Skills: A Factor of Protection Against Eating Disorders in Adolescents.” Ciência & Saúde Coletiva 20, no. 11: 3495–3508. 10.1590/1413-812320152011.18362014. 26602727

[erv70056-bib-0055] Van Huysse, J. L. , K. Smith , K. A. Mammel , N. Prohaska , and R. D. Rienecke . 2020. “Early Weight Gain Predicts Treatment Response in Adolescents With Anorexia Nervosa Enrolled in a Family‐Based Partial Hospitalization Program.” International Journal of Eating Disorders 53, no. 4: 606–610. 10.1002/eat.23248. 32092177

[erv70056-bib-0056] Warne, N. , J. Heron , B. Mars , et al. 2023. “Emotional Dysregulation in Childhood and Disordered Eating and Self‐Harm in Adolescence: Prospective Associations and Mediating Pathways.” Journal of Child Psychology and Psychiatry 64, no. 5: 797–806. 10.1111/jcpp.13738. 36541428 PMC10152493

[erv70056-bib-0057] Watson, H. J. , T. Joyce , E. French , et al. 2016. “Prevention of Eating Disorders: A Systematic Review of Randomized, Controlled Trials.” International Journal of Eating Disorders 49, no. 9: 833–862. 10.1002/eat.22577. 27425572

[erv70056-bib-0058] Westwood, H. , and K. Tchanturia . 2017. “Autism Spectrum Disorder in Anorexia Nervosa: An Updated Literature Review.” Current Psychiatry Reports 19, no. 7: 41. 10.1007/s11920-017-0791-9. 28540593 PMC5443871

